# Supplementary material for: Association of overweight/obesity and insulin resistance with activation of circulating innate lymphoid cells in women after gestational diabetes mellitus
Source: Front Immunol. 2025 Mar 10;16:1559326. doi: 10.3389/fimmu.2025.1559326 (PMC11931157; doi:10.3389/fimmu.2025.1559326)
Supplement: Supplementary file 2 [file Table1.pdf]

Table 1 Follow-up characteristics of study participants at Visit 3

Group	Control	GDM	GDMi	***Adjusted p*-value (Ctrl. vs. GDM)**	***Adjusted p*-value (Ctrl. vs. GDMi)**	***Adjusted p*-value (GDM vs. GDMi)**
No. of subjects	45	33	42			
Age (years)	39.62 ± 4.17	39.42 ± 3.99	40.52 ± 4.21	0.833	0.319	0.254
BMI (kg/m^2)	23.92 ± 4.24	22.93 ± 3.47	26.38 ± 5.77	0.277	0.026	0.003
Waist circumference (cm)	78.4 ± 8.78	77.24 ± 7.59	83.98 ±11.32	0.544	0.012	0.004
Fat mass (kg)	21.63 ± 8.29	18.74 ± 6.79	25.72 ± 9.98	0.105	0.041	0.001
HbA1c (%)	5.18± 0.29	5.33 ± 0.34	5.41 ± 0.31	0.035	0.000	0.321
HDL cholesterol (mg/dl)	67.58 ± 13.74	67.30 ± 15.38	62.48 ± 13.69	0.934	0.086	0.155
LDL cholesterol (mg/dl)	99.24 ± 28.06	95.45 ± 25.45	102.50± 29.62	0.541	0.599	0.280
Triglycerides (mg/dl)	81.22 ± 31.67	70.82 ± 33.62	103.17 ± 56.20	0.166	0.026	0.004
TSH (uIU/ml)	1.93 ± 0.98	1.65 ± 0.75	1.86 ± 0.99	0.176	0.756	0.309
Leukocytes (10^9/L)	5.38 ± 1.12	5.27 ± 1.13	6.23 ± 1.51	0.664	0.003	0.003
Glucose metabolism at Visit 3 (%)						
NGT	37 (82.2)	23 (69.7)	23 (57.2)			
IFG	7 (15.6)	5 (15.1)	9 (21.4)			
IGT	1 (2.2)	3 (9.1)	4 (9.5)			
IFG+IGT	0 (0)	2 (6.1)	5 (11.9)			
T2DM	0 (0)	0 (0)	0 (0)			

Data are presented as means (± standard deviation). Adjusted p-values were obtained from post-hoc tests adjusted for multiple comparisons following a one-way analysis of variance (ANOVA) or Kruskal-Wallis test. Visit 3 (58-66 months postpartum). GDM, gestational diabetes mellitus. BMI, body mass index. IFG, impaired fasting glucose. IGT, impaired glucose tolerance. NGT, normal glucose tolerance.
